# The risk of cataractogenesis after gamma knife radiosurgery: a nationwide population based case-control study

**DOI:** 10.1186/s12886-017-0435-1

**Published:** 2017-04-04

**Authors:** Cheng-Loong Liang, Po-Chou Liliang, Tai-Been Chen, Huan-Chen Hsu, Fu-Cheng Chuang, Kuo-Wei Wang, Hao-Kuang Wang, San-Nan Yang, Han-Jung Chen

**Affiliations:** 1 Department of Neurosurgery, E-Da Hospital, I-Shou University School of Medicine, 1, E-Da Road, Yan-Chou District, Kaohsiung, 824 Taiwan; 2 Department of Medical Imaging and Radiological Sciences, I-Shou University, Kaohsiung, Taiwan; 3 Department of Ophthalmology, E-Da Hospital, Kaohsiung, Taiwan; 4 Department of Radiation Oncology, E-Da Hospital, Kaohsiung, Taiwan; 5 Department of Pediatrics, E-Da Hospital, I-Shou University School of Medicine, Kaohsiung, Taiwan

**Keywords:** Cataractogenesis, Gamma knife radiosurgery, Lens, Computed tomography, Cerebral angiography

## Abstract

**Background:**

Medical radiation is considered a factor responsible for cataractogenesis. However, the incidence of this ophthalmologic complication resulting from gamma knife radiosurgery (GKRS) has not yet been reported. The present study aimed to determine the risk of cataractogenesis associated with radiation exposure from GKRS.

**Methods:**

This study used information from a random sample of one million persons enrolled in the nationally representative Taiwan National Health Insurance Research Database. The GK group consisted of patients who underwent GKRS between 2000 and 2009. The non-GK group was composed of subjects who had never undergone GKRS, but who were matched with the case group for time of enrollment, age, sex, history of coronary artery disease, hypertension, and diabetes.

**Results:**

There were 277 patients in the GK group and 2770 matched subjects in the non-GK group. The GK group had a higher overall incidence of cataracts (10.11% vs. 7.26%; crude hazard ratio [cHR], 1.59; 95% CI, 1.07–2.36; adjusted hazard ratio [aHR], 1.25; 95% CI, 0.82–1.90) than the non-GK group. Patients who had undergone computed tomography and/or cerebral angiography (CT/angio) studies had a higher risk of developing cataracts than those who did not (10.82% vs. 6.64%; cHR, 1.74; 95% CI, 1.31–2.30; aHR, 1.65; 95% CI, 1.22–2.23). The age group between 30 and 50 years had the highest risk of cataractogenesis in both the GK and CT/angio groups (cHR, 3.50; 95% CI, 1.58–7.72; aHR, 2.43; 95% CI, 1.02–5.81; cHR, 2.96; 95% CI, 1.47–5.99; aHR, 2.27; 95% CI, 1.05–4.93, respectively).

**Conclusions:**

Radiation exposure due to GKRS and CT/angio study may be independently associated with increased risk of cataractogenesis. We suggest routine dosimetry measurement of eye lens and proper protection for patients with benign lesions during GKRS. Regular follow-up imaging studies should avoid the use of CT/angio, and particular care should be taken in the 30–50-year-old age group, due to their significantly increased risk of cataract formation.

## Background

Over the past few decades, gamma knife radiosurgery (GKRS) has been increasingly used for intracranial lesions [1–4]. In Taiwan, the up-to-date official information indicates that there are seven gamma knife units and about 1100 patients undergo GKRS every year. Although patients who undergo GKRS account for only 6–10% of all neurosurgical patients, more than 50% of patients are diagnosed with benign lesions, including benign brain tumors, vascular lesions, and trigeminal neuralgia. Unlike for patients who have undergone conventional radiation for the malignancies, the patients with benign brain lesions who have undergone GKRS have a long life expectancy. Thus, as the number of patients undergoing GKRS is increasing, the adverse effects of the related radiation should elicit serious concern.

The hazards of radiation to the human body, such as an increased risk of cancer, have been well established by many large-scale studies on atomic bomb survivors [5]. The lenses of the eyes are the organs most sensitive to radiation injury, because of their superficial location and direct contact with the radiation beam. The relationship between cataract formation and cumulative doses of ionizing radiation has also been documented in epidemiologic studies from atomic bomb survivors [6, 7]. The International Commission on Radiologic Protection (ICRP) has published threshold values resulting in detectable lens opacities of 5 Sv for fractionated or protracted exposure and 0.5–2.0 Sv for single brief exposures [8, 9]. More recently, on the basis of the current data, the ICRP 2011 has updated the threshold radiation dose for the lens of the eye: deleterious lens opacities are induced when the absorbed dose is about 0.5 Gy. In the recommendations for occupational exposure, the equivalent dose limit for the lens of the eye should be 20 mSv per year, averaged over a period of 5 years, with exposure not exceeding 50 mSv in any single year [10]. Many recent studies have discussed radiation-induced cataracts among workers in radiology departments or among patients who have undergone repeated computed tomography (CT) [11–14]. Furthermore, because of the increasing numbers of patients undergoing GKRS and subsequent CT and/or cerebral angiography (CT/angio) for imaging studies, medical radiation-induced cataracts, particularly those resulting from GKRS and CT/angio, requires further attention. This population-based case-control study was conducted using the Taiwan National Health Insurance Research Database (NHIRD) to investigate the association between cataract development and radiation exposure from GKRS and the subsequent CT/angio imaging studies.

## Methods

### Database

Virtually all (97.11%) of the inhabitants of Taiwan (21,869,478 of 22,520,776 population by the end of 2002) are enrolled in the National Health Insurance program, which has been operating since 1995 [13, 15]. The NHIRD at the National Health Research Institutes (NHRI) in Miaoli, Taiwan, is in charge of the entire National Health Insurance claims database and has published numerous extracted datasets for researchers. For instance, the NHRI has released cohort datasets comprising 1,000,000 randomly sampled individuals who were alive in 2000 and has collected all of the records of these individuals from 1995 onward. The database is confirmed by the NHRI as being representative of the Taiwanese population. It is also one of the largest nationwide population-based databases in the world [15]. In this cohort dataset, each patient’s original identification number has been encrypted for confidentiality. Of note, the encrypting procedure is consistent, such that it is possible to link claims from the same patient within the NHIRD datasets. These comprise secondary, de-identified data, which is released to the public for research. This study was approved by the institutional review board of E-Da Hospital (No. EMRP-105-036).

### Subjects

**Fig. 1:**
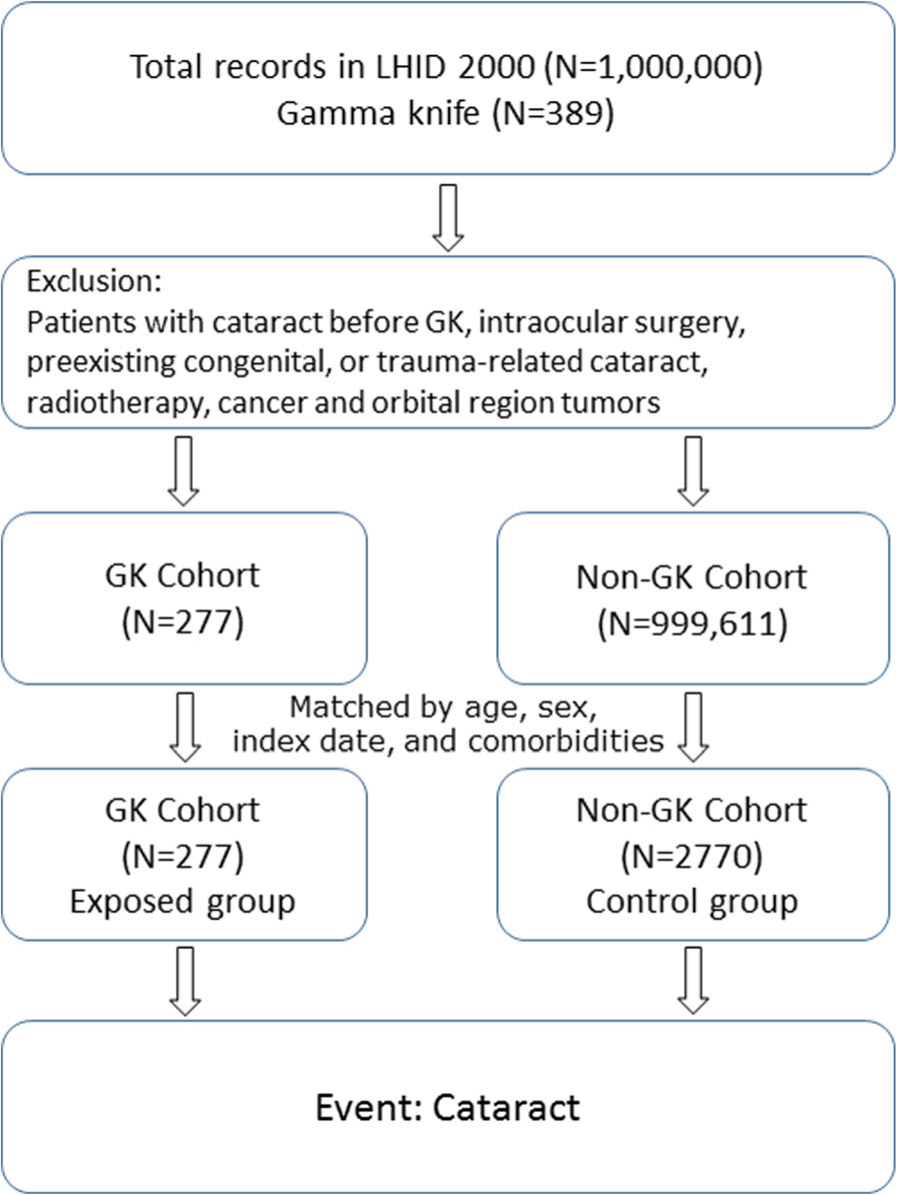
Study flow chart. LHID: Longitudinal National Health Insurance Database; GK: gamma knife radiosurgery

This study comprised two groups of individuals. The GK group was composed of patients who had undergone GKRS (based on the procedure code 37029B). The patients were identified from the longitudinal health insurance databases of the NHIRD. The data subset is composed of 1,000,000 randomly sampled beneficiaries and encompass the period 2000–2009. Among the GKRS code registered on the NHIRD, patients with brain lesions who underwent GKRS were assumed to have benign brain tumors, vascular abnormalities, including arteriovenous malformation, dural arteriovenous fistula, and cavernous angiomas, and trigeminal neuralgia. The non-GK group was composed of age-, sex-, and comorbidity-matched subjects in the NHIRD who had never undergone GKRS, on enrolment or after. Comorbidities that were included to control the confounding risk factors were a history of coronary artery disease (ICD-9-CM codes 410.xx–414.xx), diabetes mellitus (ICD-9CM codes 250.xx), and hypertension (ICD-9-CM codes 401.xx–405.xx). In both groups, subjects who underwent intraocular surgery via coded procedures, or who had preexisting, congenital, or trauma-related cataracts (ICD-9-CM codes 366.xx) before enrollment were excluded. Because cataracts are not rare complications in patients with radiotherapy, those with ocular and orbital region tumors (ICD-9-CM codes 190.x) and intracranial malignancies (ICD-9-CM codes 191.x) were also excluded to reduce the confounding factor of radiotherapy. The medical radiation from CT/angio is of concern and is not uncommon in patients who have undergone GKRS; thus, CT/angio for brain lesions was included to control for the confounding risk factor of a history of head and neck CT/angio. All patients were followed up until the study endpoint or December 31, 2009. In addition, age is an important factor in cataractogenesis [16, 17]; therefore, subjects were divided into four age groups: under 30-, 30–50-, 50–70-, and over 70-years-old to analyze the effect of aging. Figure 1 shows the study flow.

### Cataract occurrence

The endpoint of the study was the first occurrence of cataract. In this database, the ICD-9-CM codes for cataract (ICD-9-CM codes 366.xx) did not change throughout the whole follow-up period (2000–2009), ensuring the consistency of the disease registry. Subjects who met any of the following two criteria were identified as having cataract: patients who underwent cataract extraction surgery, as identified by procedure codes in the NHIRD; or patients who had at least two clinical visits coded as ICD-9-CM 366.xx combined with a therapeutic prescription for cataracts [13].

### Statistical analysis

All data were expressed as frequency (percentage) or mean (± SD). Parametric continuous data were compared between the GK and non-GK groups by unpaired Student t-test, whereas categorical data were compared using the chi-square test and Fisher exact test, as appropriate. Freedom from cataract was assessed using Kaplan-Meier analysis, and significance determined using the log-rank test. The disease-free time was calculated from the date of enrollment to the date of first diagnosis of cataract. Multivariate regression analysis was conducted using Cox proportional hazard regression analysis to evaluate whether GKRS, age, and CT/angio were independent factors associated with increased risk of cataract. All *p*-values less than 0.05 were considered significant. All data management and calculations of hazard ratios (HRs) were performed using SAS version 9.4 (SAS Institute, Cary, NC, USA).

## Results

In the sample of 1,000,000 subjects, 277 subjects underwent GKRS during the study period (GK group), and 2770 matched subjects never underwent GKRS (non-GK group). There were no significant differences in age, sex, history of hypertension, diabetes mellitus, and coronary artery diseases between these two groups (Table 1).

**Table 1:** Characteristics of the study subjects

	Non-GK	GK	*P* value
Gender			1.000
Female	1400 (90.91%)	140 (9.09%)	
Male	1370 (90.91%)	137 (9.09%)	
Age			0.996
Under 30	386 (90.82%)	39 (9.18%)	
30-50	1202 (91.06%)	118 (8.94%)	
50-70	1024 (90.78%)	104 (9.22%)	
Over 70	158 (90.80%)	16 (9.20%)	
Total	2770	277	
Mean (SD)	46.3 (15.3)	46.3 (15.3)	0.993

During the follow-up period of 10 years, 28 (10.11%) patients in the GK group and 201 (7.26%) patients in the non-GK group developed cataracts (Table 2). Patients in the GK group had a higher risk of developing cataracts than did those in the non-GK group (crude HR [cHR], 1.592; 95% CI, 1.07–2.36). After considering CT/angio, GKRS did not remain significantly associated with a higher risk of cataract development (adjusted HR [aHR], 1.25; 95% CI, 0.82–1.90; Table 2).

**Table 2:** Multivariable Cox proportional hazard regression analysis estimated risk factor regarding cataractogenesis

	Non-Cataract N (%)	Cataract *N* (%)	cHR (95% C.I)	aHR (95% C.I)
GK				
No	2569 (92.74%)	201 (7.26%)	1	1
Yes	249 (89.89%)	28 (10.11%)	1.59 (1.07-2.36)*	1.25 (0.82-1.90)
CT/angio				
No	2230 (93.34%)	159 (6.66%)	1	1
Yes	588 (89.36%)	70 (10.64%)	1.74 (1.31-2.30)*	1.65 (1.22-2.23)*

*: *p* < 0.05 *cHR* crude hazard ratio, aHR adjusted hazard ratio

**Fig. 2:**
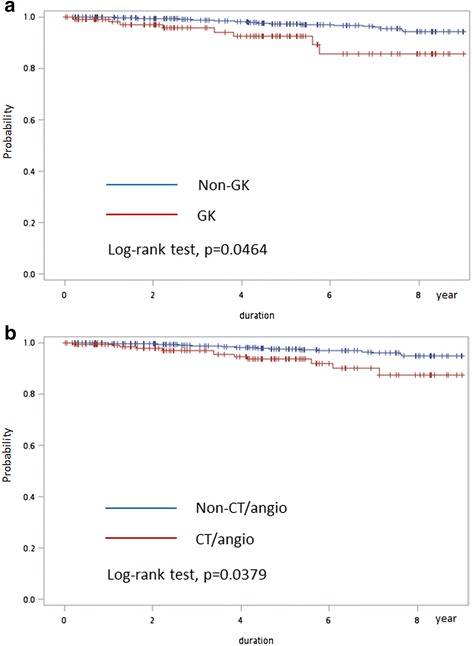
**a** Comparison of cataract-free rates between GK and non-GK cohorts in the 30–50-year-old subgroup using Kaplan-Meier model. **b** Comparison of cataract-free rates between CT/angio and non-CT/angio cohorts in the 30–50-year-old subgroup using Kaplan-Meier model

To elucidate the relationship between age and cataract risk after GKRS, age studies were performed by further stratifying subjects into four subgroups (under 30, 30–50, 50–70, over 70-years-old) to compare the associated risk of cataract accordingly. The incidence of cataract increased gradually with increasing age (0, 6.78%, 14.42%, and 31.25%, respectively; Table 3). Importantly, the 30–50-years-old individuals in the GK group had a significantly higher incidence of cataractogenesis than those in the non-GK group (cHR, 3.50; 95% CI, 1.58–7.72; aHR, 2.43; 95% CI, 1.02–5.81). During the follow-up period, the non-GK group in the 30–50-years-old subgroup had a significantly higher cataract-free survival according to the log-rank test of Kaplan-Meier survival analyses of cataract incidence (Fig. 2a).

**Table 3:** Multivariable Cox proportional hazard regression analysis estimated risk of cataractogenesis in the subjects with or without GK and with or without CT/angio in the different age groups

Age group (year-old)		Non- Cataract N (%)	Cataract N (%)	cHR (95% C.I)	aHR (95% C.I)
Under 30	GK				
	No	385 (99.74%)	1 (0.26%)	1	
	Yes	39 (100.00%)	0 (0.00%)	………..	
	CT/angio				
	No	349 (99.71%)	1 (0.29%)	1	
	Yes	75 (100.00%)	0 (0.00%)	……….	
30-50	GK				
	No	1176 (97.84%)	26 (2.16%)	1	1
	Yes	110 (93.22%)	8 (6.78%))	3.50 (1.58-7.72)*	2.43 (1.02-5.81)*
	CT/angio				
	No	1084 (98.01%)	22 (1.99%)	1	1
	Yes	202 (94.39%)	12 (5.61%)	2.96 (1.47-5.99)*	2.27 (1.05-4.93)*
50-70	GK				
	No	883 (86.23%)	141 (13.77%)	1	
	Yes	89 (85.58%)	15 (14.42%)	1.27 (0.74-2.16)	
	CT/angio				
	No	725 (86.83%)	110 (13.17%)	1	
	Yes	247 (84.30%)	45 (15.70%)	1.22 (0.86-1.72)	
Over 70	GK				
	No	125 (79.11%)	33 (20.89%)	1	
	Yes	11 (68.75%)	5 (31.25%)	2.21 (0.86-5.71)	
	CT/angio				
	No	72 (73.47%)	26 (26.53%)	1	
	Yes	64 (84.21%)	12 (15.79%)	0.60 (0.30-1.19)	

*: *p* < 0.05 *cHR* crude hazard ratio, *aHR* adjusted hazard ratio

CT/angio study was also significantly associated with a higher risk of cataract development (cHR, 1.74; 95% CI, 1.31–2.30; aHR, 1.65; 95% CI, 1.22–2.23). When we compared the CT/angio and non-CT/angio group, the 30–50-year-old individuals were also found to have a higher incidence of cataractogenesis (cHR, 2.96; 95% CI, 1.47–5.99; aHR, 2.27; 95% CI, 1.05–4.93). During the follow-up period, the non-CT/angio group in the 30–50-year-old subgroup had a significantly higher cataract-free survival based on log-rank testing of Kaplan-Meier survival analyses of cataract incidence (Fig. 2b).

## Discussion

Cataracts, defined as lens opacity, are the most frequent cause of visual impairment worldwide [16]. Major risk factors include ocular trauma, intraocular surgery, diabetes mellitus, corticosteroid use, and radiation exposure [16, 18]. In the past few decades, excessive doses of ionizing radiation have also been documented to induce opacities in eye lenses [19, 20]. Recent studies from atomic bomb survivors, Chernobyl clean-up workers, and residents of radiationcontaminated buildings have shown that the threshold radiation dose for inducing lens opacity is lower than that previously reported [6, 21, 22]. Dose estimation due to inadequate data in such populations is difficult, whereas subsequent calculated risks in these epidemiologic studies may have limited validity. Aside from epidemiologic studies, several smaller studies that examined cataract in relation to self-reported exposure to CT revealed either no association or an association for posterior subcapsular cataracts at relatively low doses of radiation (0.1–0.3 Gy) [23].

Globally, the cumulative number of GKRS procedures reached 1,000,000 in 2016. In Taiwan, an increasing number of GKRS is being performed (1100 GKRS procedures in 2015) and more than 50% of these patients were diagnosed with benign intracranial lesions. The present study demonstrated that patients who have undergone GKRS have a significantly higher risk of developing cataracts than those who have never undergone GKRS. Subsequent analysis showed that CT/angio studies are also associated with increased risk of cataract. The 30–50-years-old subgroup has a significantly higher incidence of cataractogenesis in both GK and CT/angio analysis.

The present study used a single data subset, composed of 1,000,000 randomly sampled beneficiaries, which included approximately 300 subjects who had undergone GKRS procedures, along with the corresponding information. We retrospectively compared these data with an age-, sex-, and comorbidity-matched non-GK group, and found a trend for an increased rate of cataract incidence with GKRS. Cox proportional hazard regression model analysis showed that radiation exposure from GKRS in the 30–50-years-old subgroup is independently associated with an increased risk of developing cataracts.

The dose of radiation to eye lenses during GKRS may be underestimated. According to our previous study, the radiation dose to which eye lenses of patients who underwent GKRS for trigeminal neuralgia is exposed may reach 1.1 Gy in the absence of proper lens protection, and may decrease to 0.2 Gy when lenses are appropriately protected [24]. This indicates that routine dosimetry measurement of eye lens and proper protection should be encouraged for patients with benign lesions undergoing GKRS.

In Taiwan, a large number of CT procedures is performed (1,268,921 CT procedures in 2008), which contributed 50.8% of the annual collective and average effective doses of medical radiation exposure [13, 25]. The typical effective dose of head CT is estimated to be 1–2 mSv for a diagnostic single-detector CT. The lens of the eye receives absorbed doses, up to 50 mGy, during head CT examinations [26]. The effective dose of diagnostic cerebral angiography is estimated to be 10 mSv [27]. The lens of the eye receives absorbed considerably higher doses, up to 500 mGy, during cerebral angiography. The eye lens is under direct radiation exposure during CT/angio examinations. Most patients undergo CT/angio examinations without eye shields, because artifacts caused by eye protective shields may affect the quality of images. The results of the present study indicate that the risk of radiation-induced cataracts during CT/angio examination is higher than may be previously expected, and should not be ignored. However, it is difficult to identify the exact radiation dose during CT/angio examinations in the nationwide population study. Suggestions such as low-dose CT using iterative reconstruction and the use of protective shielding materials with minimal artifacts should therefore be encouraged. Because of rapid progress in the development of high-technology CT/angio machines, the new CT/angio would progressively decrease the radiation to human subjects. Regardless of the doses of radiation exposure, it is universally agreed that the less radiation received, the lower risk. For this reason, it is considered prudent to attempt to maintain exposures as low as reasonably achievable. Therefore, the authors suggests MRI and MRA studies in regular follow-up imaging studies.

Consistent with previous reports of various study populations, increasing age was also found to be a significant risk factor for all types of cataracts and cataract surgery, even after adjusting for all major covariates. The effects of aging may reflect contributions from several factors, such as the accumulation of damage from the environment, the deterioration of defense and repair mechanisms, and genetic predisposition. In the present study, the cataract occurrence rate increased gradually with increasing of age, with the significantly high incidence occurring in the 30–50-year-old subgroup; this incidence was found to be higher in the GK group than the non-GK group and in the CT/angio than the non-CT group. The explanation may be the cataract occurrence rate is typically low in the 30–50-year-old subgroup, but medical radiation due to GKRS and sebsequent CT/angio could induce cataractogenesis.

Patients with intracranial malignancies were excluded from the study. Given the increasing survival for patients with metastatic cancer, and the high percentage worldwide of such patients undergoing GKRS, the risk of cataractogenesis in brain metastasis patients might be an issue of concern [28]. However, the risk of cataractogenesis in these patients would be of relatively little concern compared to the benefits of GKRS. The cataract surgery is a well-established and generally safe procedure, this increased risk may be worth it to patients if they are cured or controlled of their malignant lesions with GKRS.

The current study has limitations. First, the number of subjects who underwent GKRS (*N* = 277) is a small proportion of the 1,000,000 individuals sampled from the NHIRD. We have performed analysis on another sampled population of 1,000,000 and found very similar results as those presented here (data not shown). Second, the data from the NHIRD can only provide information about the frequency of GKRS. The target area varies with different disease entities and GK protocols, and the scattered radiation dose cannot be determined exactly in our study. The types, locations, volumes, and prescription doses of lesions which may influence the radiation exposure of eye lens were not documented in the NHIRD. Third, corticosteroid and multivitamin use were also not assessed in this study, because the miscellaneous corticosteroid and multivitamin prescriptions in Taiwan are difficult to extract in the NHIRD. Fourth, we did not evaluate the risk associated with education, body weight, and smoking, which are not documented in the NHIRD. Despite these limitations, the present study is the first nationwide population-based study to focus on the association between cataract and GKRS. The results provide valuable evidence of the medical radiation hazards to the lens of the human eye.

## Conclusions

In conclusion, this 10-year retrospective nationwide population-based study, using the NHIRD in Taiwan, shows that GKRS and CT/angio may be independently associated with a significantly increased risk of cataract occurrence in the 30–50-year-old age group. We suggest routine dosimetry measurement of eye lens and proper protection for patients with benign lesions during GKRS. Regular follow-up imaging studies should avoid the use of CT/angio, and particular care should be taken in the 30–50-year-old age group.

## Abbreviations

CT/angio: Computed tomography and/or cerebral angiography
GK: Gamma knife
GKRS: Gamma knife radiosurgery
ICRP: International Commission on Radiologic Protection
NHIRD: National Health Insurance Research Database
NHRI: National Health Research Institutes
